# A Web-Based Institutional DICOM Distribution System with the Integration of the Clinical Trial Processor (CTP)

**DOI:** 10.1007/s10916-014-0186-y

**Published:** 2015-03-03

**Authors:** K. Y. E. Aryanto, A. Broekema, R. G. A. Langenhuysen, M. Oudkerk, P. M. A. van Ooijen

**Affiliations:** 1 Department of Radiology, Center for Medical Imaging - North East Netherlands (CMINEN), University of Groningen, University Medical Center Groningen, Hanzeplein 1, Postbus 30001, 9700 RB Groningen, The Netherlands; 2 Department of Radiology, University Medical Center Groningen, Groningen, The Netherlands; 3 Rogan B.V., Huis ter Heide, Utrecht, The Netherlands

**Keywords:** DICOM distribution system, Web based system, Clinical trial processor, Vendor-neutral system

## Abstract

To develop and test a fast and easy rule-based web-environment with optional de-identification of imaging data to facilitate data distribution within a hospital environment. A web interface was built using Hypertext Preprocessor (PHP), an open source scripting language for web development, and Java with SQL Server to handle the database. The system allows for the selection of patient data and for de-identifying these when necessary. Using the services provided by the RSNA Clinical Trial Processor (CTP), the selected images were pushed to the appropriate services using a protocol based on the module created for the associated task. Five pipelines, each performing a different task, were set up in the server. In a 75 month period, more than 2,000,000 images are transferred and de-identified in a proper manner while 20,000,000 images are moved from one node to another without de-identification. While maintaining a high level of security and stability, the proposed system is easy to setup, it integrate well with our clinical and research practice and it provides a fast and accurate vendor-neutral process of transferring, de-identifying, and storing DICOM images. Its ability to run different de-identification processes in parallel pipelines is a major advantage in both clinical and research setting.

## Introduction

Over the last decade, Picture Archiving and Communication Systems (PACS) evolved from a predominantly radiological service into the fundament of the image management system utilized throughout the entire healthcare enterprise for clinical practice and research [1–4]. It integrates multimedia, information, and communication technologies, providing practically unlimited amounts of data for patient care, research and teaching [5].

PACS integration with other information systems has made medical image distribution to play an important role in the entire clinical process including the interpretation, processing and analysis of imaging data [6, 7]. Distribution of images and information to clinicians in a timely manner is therefore required to facilitate clinical work-up. Although current high-speed networks allow exchange or transfer of data from one node to another at acceptable speed, optimization of the processing of digital images during the distribution is also a prerequisite to meet the clinical and research needs.

The Digital Imaging and Communication in Medicine (DICOM) standard has been widely adopted for recording and sharing of digital medical images [8]. A DICOM image consists of a header with patient and exam data firmly connected with the image pixel data in a single file. The DICOM header contains a large amount of information related to the pixel data it belongs to, including identifiable information about the patient, the study, and the institution. Because of this inclusion of possible sensitive data, de-identification of DICOM images is often required in data exchange for clinical research [9, 10].

The information embedded in the DICOM data should be handled carefully to ensure the security of patients’ identity, especially when transfers are intended for a clinical trial or research. The importance of security issue had motivated research in the utilisation of health information within data exchange and the use of information system technology [11–13]. However, the process of de-identifying the data should be implemented such that it provides ease of use and simple configuration in order to facilitate all users – including in-experienced ones - to adequately fulfil the task of ensuring the patient data protection. The imaging IT infrastructure within an institution may consists of many smaller systems that support each other Of these systems, some are often not integrated within the normal workflow but operated as standalone applications thus reducing the ease of use and increasing the risk of errors and mistakes. Therefore, other than just the ease-of-use of the systems introduced to handle a certain task, the ease of integration of those systems have also become an important issue to adequately fulfill the requirements of smooth integration into the daily work processes. Therefore, besides providing an easy to use process, health institutions are also required to develop systems that are ready to be integrated into the normal workflow using current standards.

To facilitate data distribution within a hospital environment, a web-based system called RadTransceiver was built. The system was implemented as a fast and easy web-environment, providing an integrated rule-based distribution and optional de-identification of imaging data.

## Materials and methods

The server side of the RadTransceiver runs under the Microsoft Windows Server 2003 operating system. All RadTranceiver services were developed using Java [14], a multi platform general purpose object oriented programming language, with Microsoft SQL Server [15] to handle the database management system. The Graphical User Interface (GUI) was designed using PHP: Hypertext Preprocessor (PHP) [16], a general-purpose scripting language which is especially suitable for Web development and which can be embedded into HyperText Markup Language (HTML). Web-based system is deployed to enable the broader range of utilisation, the ease of use and understand, with the interfaces that suit the variations in computer literacy [17].

User rights in accessing RadTransceiver are divided into three types: superuser, managerial user and ordinary user. A superuser has unrestricted access to all features of the system. This category of users contains the administrators who are responsible to manage and supervise the whole system and take actions over unwanted activities or erroneous usage of the system. Managerial users have access to the managerial features of the system to view all image processes and their status. Ordinary users have limited rights, can only view and access their own initiated processes and have no access to the critical features of the system or information which belongs to other users. Superusers and managerial users have the ability to create and make changes of the transfer rules while ordinary users are only able to perform image transfer based on the transfer rules that are related to their login credentials.

**Fig. 1:**
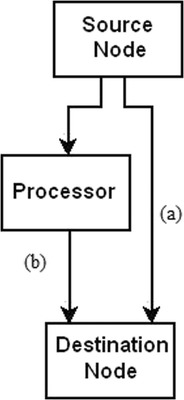
Two kinds of image transfer: **a** a direct transfers from one node to the destination node (such as from PACS to viewers); **b** a transfer through a processor before images reach the destination

There are two kinds of image transfer that could be initiated by the users. First is a direct transfer from source to destination node, for example the transfer to a viewer in the Electronic Patient Record (EPR) system. With the second type of transfer the images have to pass through a processor before the images are transferred to the destination node (Fig. 1).

To perform the processing of the images the services provided by the Clinical Trial Processor (CTP) are utilized [18]. CTP is a stand-alone DICOM Application Entity (AE) developed by the Radiological Society of North America (RSNA) [19]. It has the capability of using multiple, independently configured, pipelines for running several processes in parallel. With this flexibility and configurability of pipelines the de-identification of selected patient data can be performed using a variety of pertinent rules and regulations [20]. In our DICOM distribution system, each pipeline runs its own CTP DICOM import service. This import service consists of a Storage Service Class Provider (Storage SCP), accepting images from other DICOM nodes (e.g., PACS or modality) and putting them in a queue for further process.

**Fig. 2:**
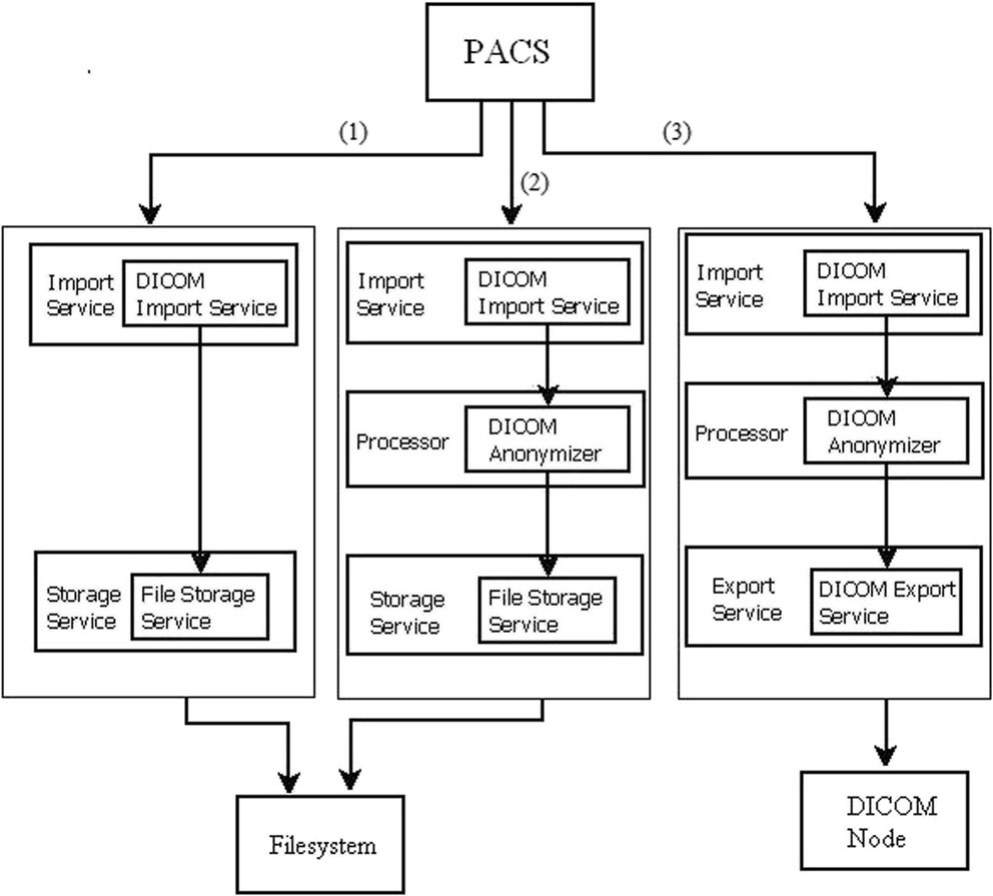
Data are transferred using one from three types of CTP pipelines set for the processor: *1*) a pipeline without de-identification scheme to a local file system; *2*) a pipeline with a de-identification process before the images are stored in a specific directory; *3*) a pipeline with a de-identification process before the images are sent to another DICOM node by the export service

In our setup, three possible pipelines are defined (Fig. 2).

1. A pipeline without de-identification sending data into a file system.
2. A pipeline with de-identification that exports the images in a specific directory or network attached storage.
3. A pipeline with de-identification sending the DICOM images to a DICOM node using the DICOM export service.

In case of transfer without de-identification, the pipeline consists of a Storage SCP as part of the Import Service followed by a Storage Service Class User (Storage SCU) as part of the Export Service. Note that a Service Class Provider (SCP) is a DICOM node or device that provides specific DICOM network services such as for instance those offered by the Storage Class defined by DICOM. A Service Class User (SCU) is the client role which makes use of DICOM Services offered by an SCP. The RadTransceiver is an Application Entity (AE) that is an SCU as well as an SCP for the DICOM Storage Service Class. An AE is a node or an application providing DICOM Services within the network. In RadTransceiver the storage service class mechanism saves images into specific folders defined in the configuration file.

When required, a pipeline with an anonymizer is configured to de-identify images before they are stored in a DICOM node. The execution of a de-identification task will trigger a service to add a record for each image to be de-identified into a confidential database, registering the identity mapping between original and de-identification ID.

A separate service monitors the amount of files being stored. When the amount of files in one task matches the amount as registered in the database, the service will mark the transfer task as a completed process.

## Results

After being logged in into RadTransceiver, a user initiates and registers the transfer of DICOM data through the web interface by entering a patient ID and selecting the appropriate images to be transferred from the PACS. DICOM transfer of the selected images to another DICOM node without de-identification is performed instantly while all other transfers are pushed into a processor service. Using the processor, images can be transferred from the PACS or modality into the predefined storage destination or sent through a process of image de-identification using a specific profile before they are stored.

The managers of RadTransceiver can create one or more user-specific module(s) related to the type of clinical research study and associate a transfer protocol for that user/study combination. The transfer protocol destination node defines how it has to be stored (file system or DICOM) and whether it has to be de-identified or not. This allows to configuring and utilizing different modules for specific tasks and users. These modules are linked to the user account and thus a user can only execute those modules assigned to his/her user profile.

**Fig. 3:**
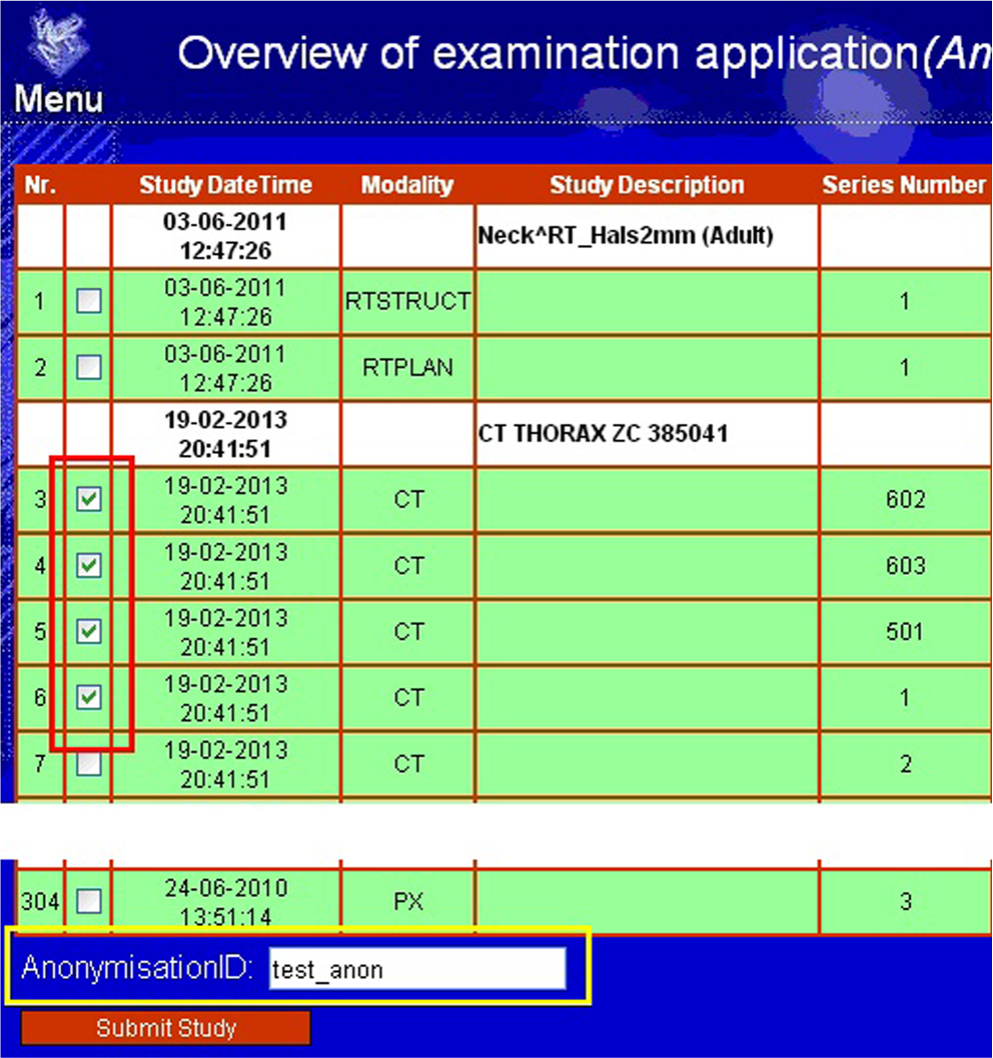
Web interface. The web interface shows the results of a search for a patient from the PACS based on the patient ID, selection of images (surrounded by the *red* rim on the left side), and entry of an anonymizationID (surrounded by the *yellow* rim) in the bottom part of the web page

The result of a patient ID based search from the PACS provides a list of selectable items of available study entities and serie within those entities in DICOM files. Date, time and study descriptions are shown for each study entity. The date and time, modality type, series number, series description, number of images, and accession number are shown for each series. The user then selects the required series by ticking the checkboxes at the beginning of the corresponding row. Further processing will be done after the submission button is clicked by the user. The de-identification ID is entered by the user using an input box provided at the bottom of the page. When no de-identification ID is provided a time-stamp based de-identification ID will be automatically generated. The search results, study entity selection and entry of identification-ID are shown in Fig. 3.

Some modules are triggered automatically from the EPR and designed to transfer data from PACS directly to the EPR without user interaction. Since EPR users can send requests of image retrieval through the system, from the image viewing process, the EPR itself can be considered as a user that can initiate image transfers from PACS.

**Fig. 4:**
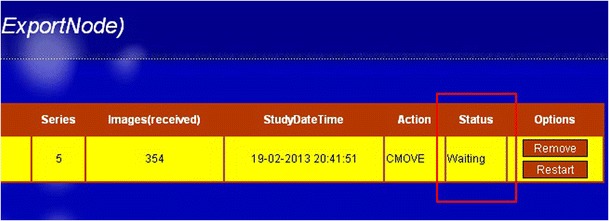
The web interface shows the selected images are queued and waiting to be processed. The status indicates that *images* are in the “waiting” list

**Fig. 5:**
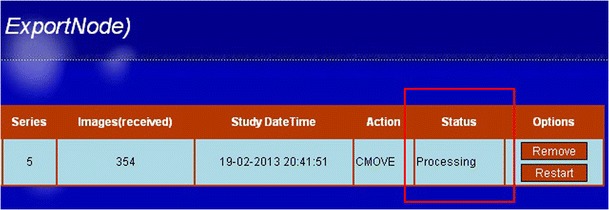
The web interface shows the selected images are being handled by the system. The status indicates that the *images* are being processed

Users can track the status of their own transfers by accessing the web interface for the associated process showing a list of jobs in ‘waiting’ (Fig. 4) and ‘processing’ (Fig. 5) state.

RadTransceiver also has a priority system that affects the order of processing of the images. Transfer processes with a higher priority always appear in the top rows of the queue and are thus handled right after the current active process has finished. When there is more than one process with the same priority they will be sorted based on their time of requests, following the ‘first in - first out’ principle.

The initiation of a de-identification task by the user will generate a mapping of the original, unique, identifier into a de-identification identifier both in the database and the lookup-table. The update on the database is required to register the transfer itself and to record the amount of images being sent to be matched in the last stage of the process. Meanwhile, the creation of id-mapping on the lookup-table will be used in the de-identification process itself. The lookup-table is updated continuously and only contains the id-mapping of active transfers. The mapping will be removed when a transfer task is finished.

**Fig. 6:**
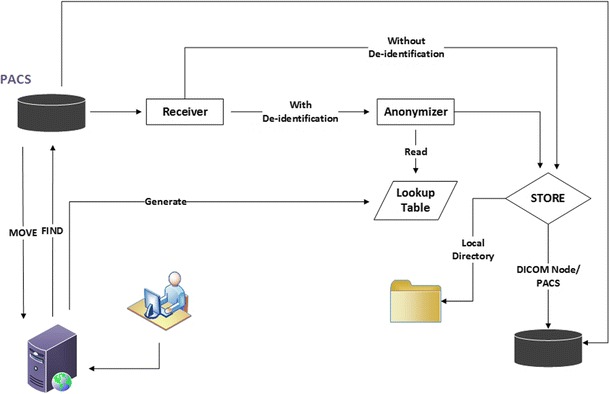
Web based DICOM distribution system with CTP integrated as its image processor

Five pipelines composed of a minimum of one DICOM import service and either a DICOM storage or export service are defined in the CTP server to provide different DICOM nodes with images according to their specific needs. With the DICOM export service set in the configuration, DICOM images received by the CTP were stored at a different DICOM node within our institution. The resulting images were all stored at the correct DICOM node without any duplication occurring. The anonymizer was set only when the related transfer required de-identification. Figure 6 shows the entire system.

**Fig. 7:**
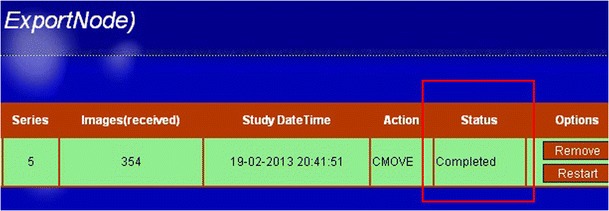
Finished task shown in the web interface. The status (surrounded by the *yellow rim*), is indicated by “completed”. The buttons next to the status are used for restarting or removing the task

Processes can be monitored by observing the status of the process in the web pages. When failures occur, the user can restart or remove the tasks using the corresponding buttons on the rightmost sisde of the task-row. Figure 7 shows the finished process in the web interface.

**Fig. 8:**
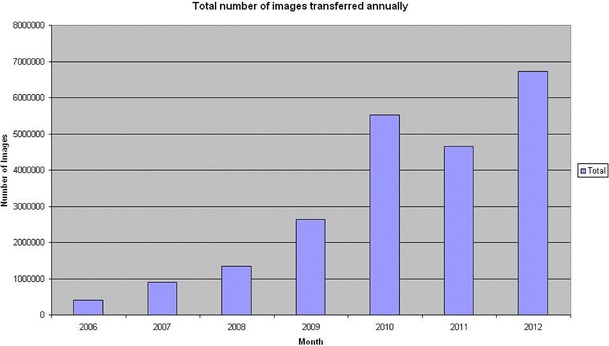
Total number of *images* which have been transferred annually since October 2006 until December 2012

**Fig. 9:**
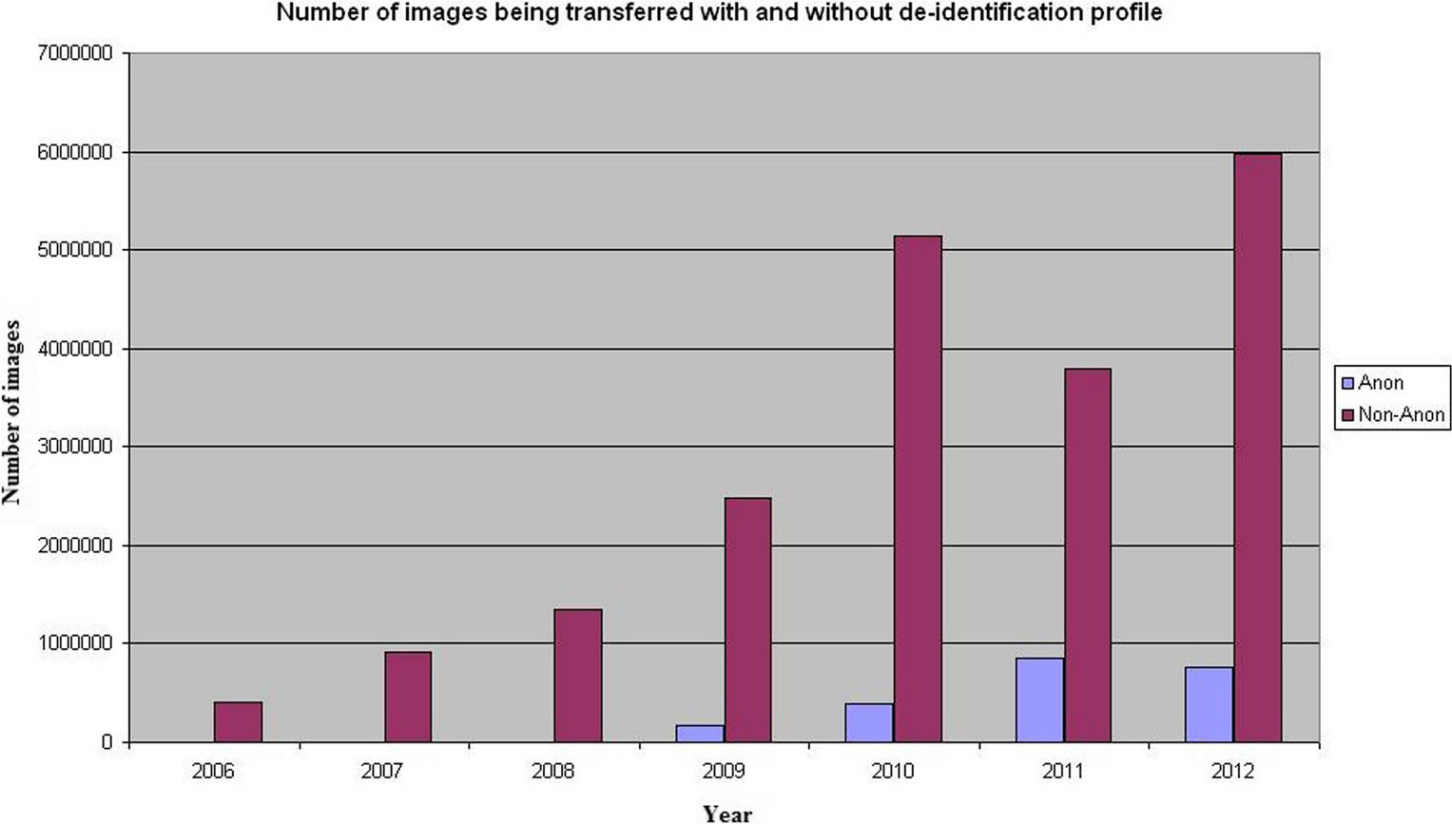
Number of *images* being transferred with and without de-identification profile

The system has transferred more than 24 millions images during 75 months of stable operation (October 2006 until December 2012). More than 2,000,000 images were transferred and de-identified in a proper manner while the rest (20,000,000) were only moved from one node to another without de-identification. Of the data transferred, 10 % was de-identified and thus used for clinical research trials or teaching, the remaining data was transferred for patient care. The data without de-identification was transferred within our institution to move older imaging data from one system (the PACS) to the other (institutional web browser). In Fig. 8 the total number of images which have been transferred within 75 months until December 2012 is shown and Fig. 9 displays the difference regarding images transferred with the identification profiles and without it. By performing multiple transfers using the system, time from request to delivery of the images was computed covering the whole process of transfer, de-identification, and data strore. The system delivers images at an average rate of four CT images per seconds (Table 1), and MR images at an average rate of eight images per seconds.

**Table 1:** Several transfers using CT and MR images, giving a rate of four CT images per second and eight MR images per second

Type	Number of images	Size (MB)	Time (Seconds)	Images per second
CT	100	52	22	5
	500	251	120	4
	1000	505	253	4
	2000	1000	460	4
MR	100	13	12	8
	1000	108	124	8
	5000	615	627	8

## Discussion

A DICOM data distribution system was developed to provide a fast and easy environment to facilitate the distribution of medical images within an institution. The use of commonly known web based technologies and the flexible processor make it easy to implement and adapt to specific use.

DICOM de-identification covers the security of patient-related data, but data trackback is still possible by retrieving the de-identification entry in the conversion table. Therefore, the access to the clinical research study information system database and conversion table is limited to authorized personnel and can be obtained through an internal network only.

The large number of images transferred without de-identification is caused by the fact that this setup is also used to facilitate the retrieval of image data into an EPR. The DICOM web-viewer integrated into our EPR is limited to about 1 year of storage. To enable easy access to older data, EPR users can automatically retrieve the historical data from their patient with one click within the EPR system. Requests through the EPR are considered as high-priority tasks and therefore, the data will immediately be transferred from PACS to the EPR.

In many institutions the production of de-identified data is performed from the PACS. Thus, it requires the use of a full PACS workstation for the researcher. This involves a costly PACS license and blocks the workstation for actual clinical reporting use for the time required for the de-identification. Alternatively, users get the non de-identified DICOM data locally and perform the de-identification process manually. This leads to the possibility of use of a wide variety of not properly managed tools and thus cannot ensure patient data and identity safety. Using RadTransceiver, the access is provided through a simple, password-protected, webpage which can be accessed from any PC in a hospital; the de-identification process is controlled and supervised centrally and is vendor neutral.

In some cases trace-back of the actual patient behind the de-identified data is required, for example in the case of incidental findings in scans made for a research study. Therefore, our de-identification method, sometimes referred to as pseudonymization, uses a lookup-table which allows tracing back the original patient information. However, only necessary data are pseudonymized while the remaining fields are made anonymous. This is the reason why CTP is used within our system; besides transferring and processing the DICOM data in proper manner to provide patient data security, integrity and confidentiality, it also provides simple yet flexible and extensive configuration of DICOM de-identification profiles.

The lookup-table consist of an ID-mapping between the original and the de-identified ID and was used by the system to record and guide the de-identification process. However, besides the replacement of the patient ID as used in the lookup-table a whole set of 50 DICOM header tags are de-identified during the de-identification process (see Table 2). These 50 tags are considered to be the possible cause of data breach when they are exposed to a third party either by the element itself or combination with other elements. All 50 elements are replaced by the de-identification ID, a blank, or a more general representation. Although this provides a proper de-identification of the DICOM header, a more comprehensive de-identification, including blanking of part of the image, should be considered in accordance to the DICOM Supplement 142 Clinical Trial De-identification profiles to ensure the full protection of patient’s private data [21].

**Table 2:** Fields in the DICOM header defined to be de-identified

Tag ID	Tag name	Tag ID	Tag name
0008,0020	StudyDate	0008,1060	NameOfPhysicianReadingStudy
0008,0021	SeriesDate	0008,1062	PhysicianReadingStudyIDSequence
0008,0022	AcquisitionDate	0008,1070	OperatorsName
0008,0023	ContentDate	0010,0010	PatientsName
0008,0024	OverlayDate	0010,0020	PatientID
0008,0025	CurveDate	0010,0021	IssuerOfPatientID
0008,002A	AcquisitionDatetime	0010,0030	PatientsBirthDate
0008,0030	StudyTime	0010,0032	PatientsBirthTime
0008,0031	SeriesTime	0010,0040	PatientsSex
0008,0032	AcquisitionTime	0010,1000	OtherPatientIDs
0008,0033	ContentTime	0010,1001	OtherPatientNames
0008,0034	OverlayTime	0010,1005	PatientsBirthName
0008,0035	CurveTime	0010,1010	PatientsAge
0008,0050	AccessionNumber	0010,1040	PatientsAddress
0008,0080	InstitutionName	0010,1060	PatientsMothersBirthName
0008,0081	InstitutionAddress	0010,2150	CountryOfResidence
0008,0090	ReferringPhysiciansName	0010,2152	RegionOfResidence
0008,0092	ReferringPhysiciansAddress	0010,2154	PatientsTelephoneNumbers
0008,0094	ReferringPhysiciansTelephoneNumber	0020,0010	StudyID
0008,0096	ReferringPhysicianIDSequence	0038,0300	CurrentPatientLocation
0008,1040	InstitutionalDepartmentName	0038,0400	PatientsInstitutionResidence
0008,1048	PhysicianOfRecord	0040,A120	DateTime
0008,1049	PhysicianOfRecordIDSequence	0040,A121	Date
0008,1050	PerformingPhysiciansName	0040,A122	Time
0008,1052	PerformingPhysicianIDSequence	0040,A123	PersonName

The export and store services provided by the processor can also be utilized for image sharing between health enterprises. De-identified images can be directly uploaded using a DICOM or Hypertext Transfer Protocol (HTTP) or stored into a specific directory of the system that will transfer images to outside the institution. Therefore, this system would be adaptable for the implementation of a large multi-centre clinical trial study. The interface of the DICOM distribution system was built using web technologies, therefore, wider access is possible. The de-identification profiles will be kept controlled since the transfer will be based on the existing modules in the system. However, going beyond the borders of the own institution would require proper protection of the database and table containing the relation between the de-identification ID and the real patient credentials. Furthermore, the system architecture and security should be considered carefully to ensure the safety of the system in delivering images with embedded patient data. For those reason, communication within our systems was still limited to one hospital only but currently in further development to enable collaborative studies involving many health institutions.

In conclusion, we have devised a vendor neutral system that supports data distribution and de-identification tasks in an easy, robust and user friendly way, providing a fast process in an easy setup environment while still being able to maintain a high level of security and stability. Its ability to run different de-identification processes in parallel pipelines accessible through a simple web interface is a major advantage in both clinical practice and clinical research setting.
